# Supplementary material for: The Gal4-Type Transcription Factor Pro1 Integrates Inputs from Two Different MAPK Cascades to Regulate Development in the Fungal Pathogen Fusarium oxysporum
Source: J Fungi (Basel). 2022 Nov 24;8(12):1242. doi: 10.3390/jof8121242 (PMC9781702; doi:10.3390/jof8121242)
Supplement: Supplementary file 1 [file jof-08-01242-s001.zip › jof-2047863-supplementary.pdf]

Supplementary Figures

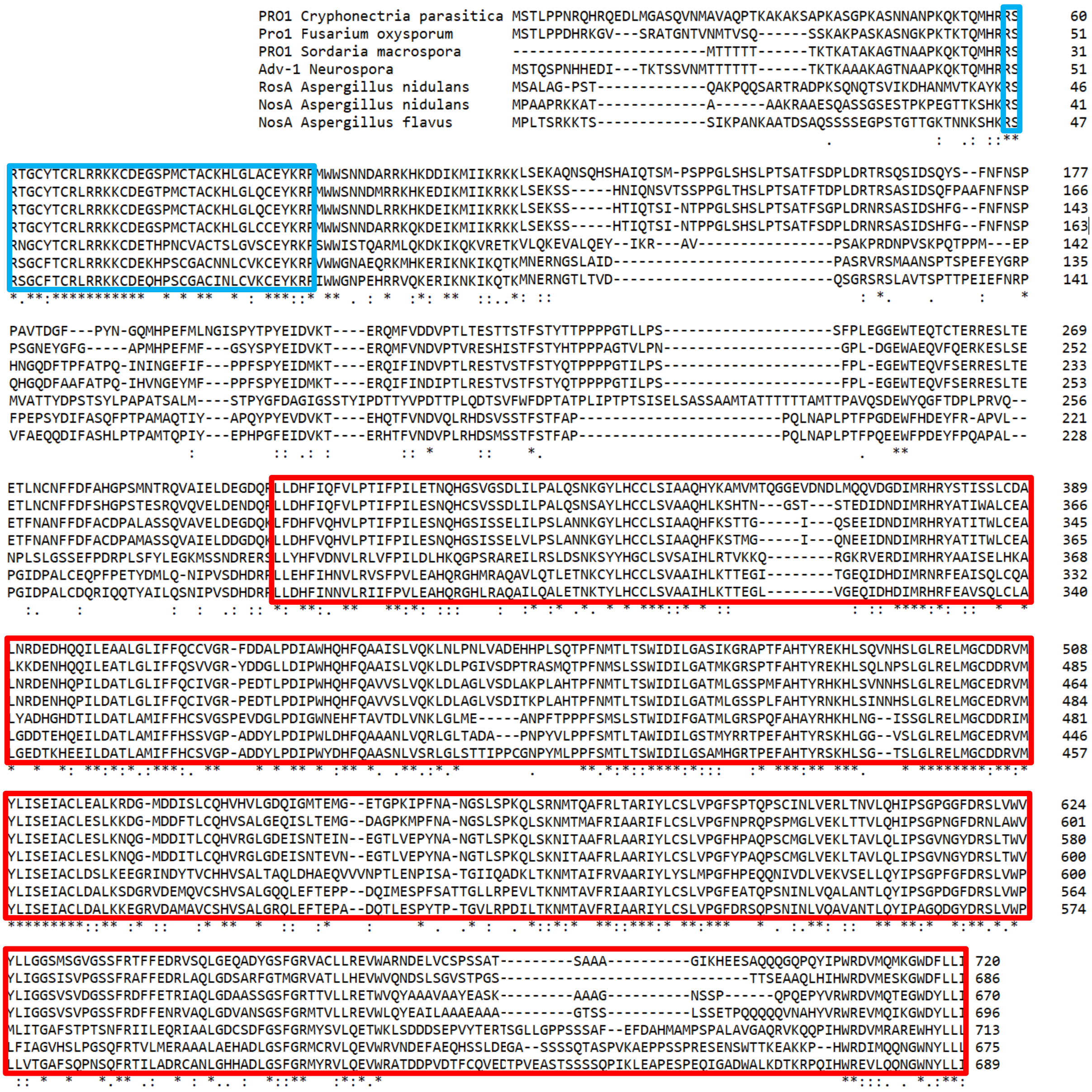

(A)

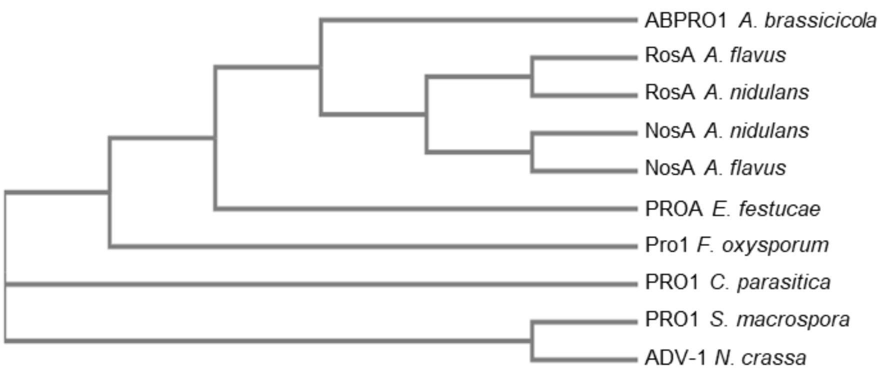

(B)

Supplementary Figure S1. Amino acid sequence alignment of the F. oxysporum Pro1 protein. (A) Sequence alignment of fungal Pro1 orthologues. The indicated full-length Pro1 protein sequences were obtained identified by BLASTP search in the

genome database of the National Center for Biotechnology Information, using the *Sordaria macrospora* k-hell PRO1 amino acid sequence (NCBI database: XP_003351793.1). Amino acid sequences of the different Pro1 proteins were aligned using Clustal W [38] and manually inspected. Asterisks, double dots and single dots indicate highly, less highly and moderately conserved residues. The GAL4-like Zn_2Cys_6 binuclear cluster DNA-binding domain is boxed in blue and the fungal specific transcription factor domain in red. (B) Phylogenetic tree of Pro1 orthologues based on sequence alignment of the complete predicted amino acid sequences was generated using the Clustal W Modelgenerator algorithm.

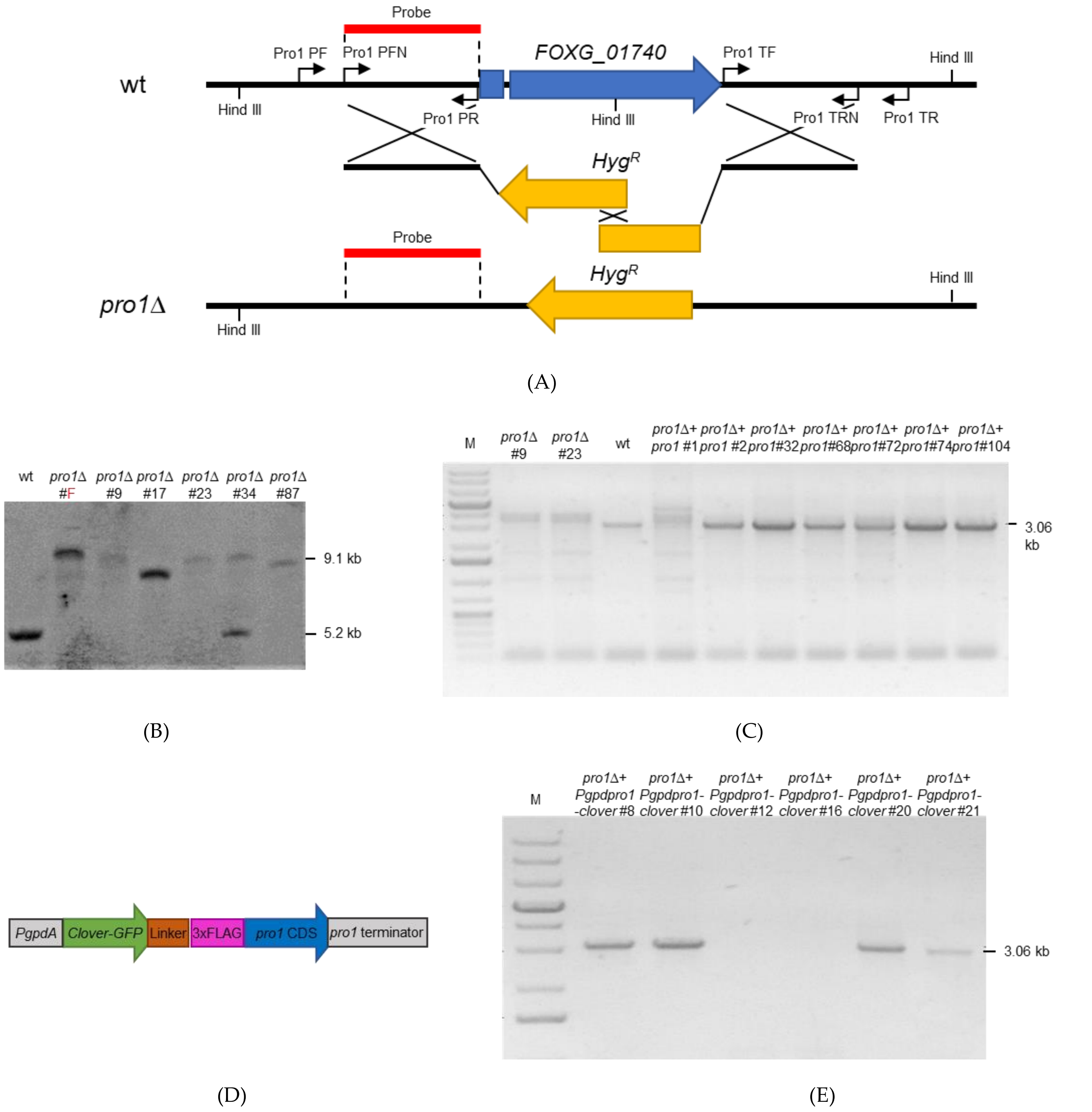

Supplementary Figure S2. Targeted replacement of the *pro1* gene in *F. oxysporum*. (A) Physical maps of the *F. oxysporum pro1* locus and of the split-marker gene replacement constructs obtained by fusion PCR. Relative positions of restriction sites, PCR primers and probe used in Southern blot are indicated. *Hyg^R*, *hygromycin* resistance gene. (B) Southern blot analysis of putative *pro1Δ* transformants. Genomic DNA of the wild type (wt) strain and six independent hygromycin resistant transformants was treated with

*Hind*III, separated on a 0.7% agarose gel, transferred to a nylon membrane and hybridized with a DNA probe corresponding to the 5′ flanking region of *pro1* indicated in (A). Molecular sizes of the hybridizing bands are indicated to the right. (C) Agarose gel electrophoresis of PCR products obtained using the primer pair CloverPro1 and Pro1compRnest with genomic DNA extracted from the indicated neomycin-resistant transformants. (D) Schematic representation of the *pro1*Δ *PgpdA-pro1-GFP* construct. (E) Agarose gel electrophoresis of PCR products obtained using the primer pair Gpda5 and Pro1Rev with genomic DNA extracted from the indicated neomycin resistant transformants. M, molecular size markers.

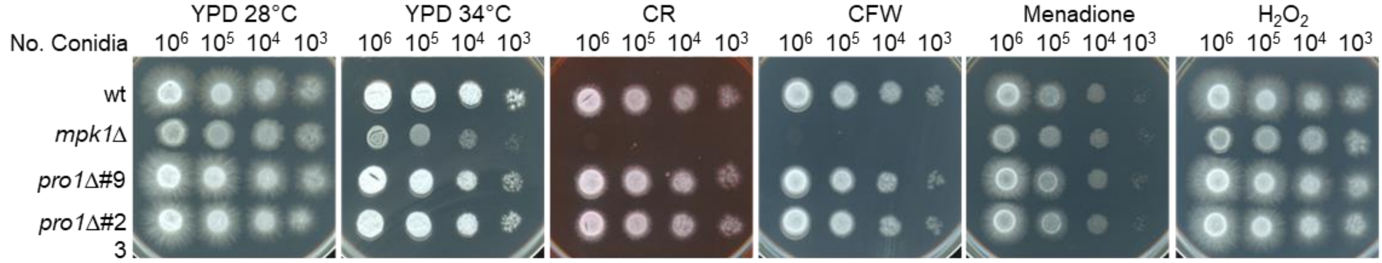

(A)

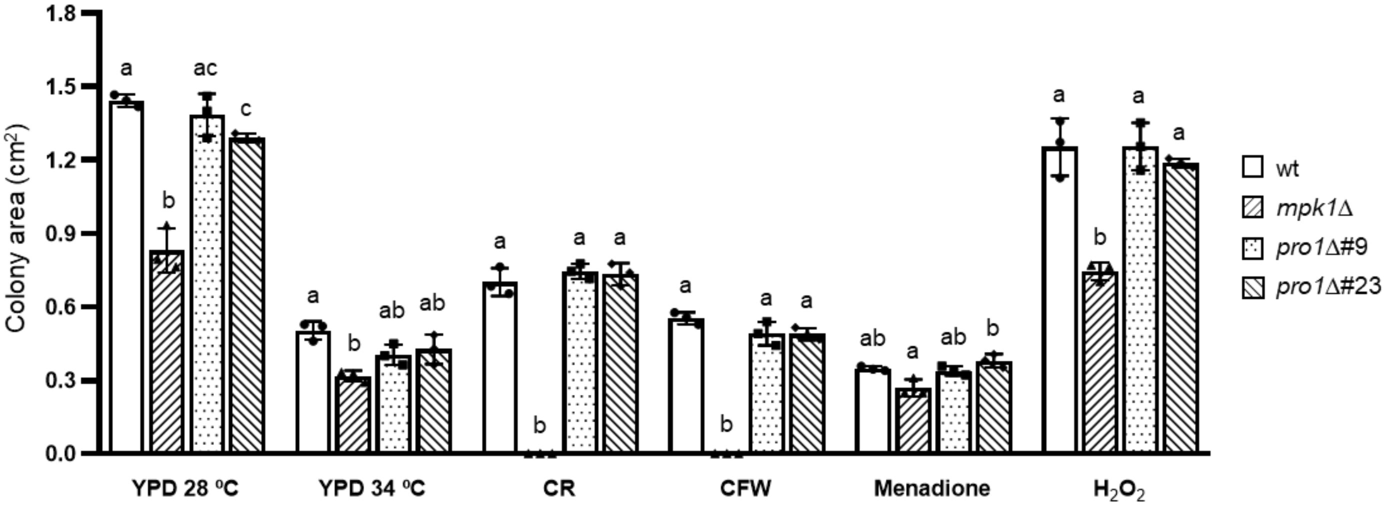

(B)

Supplementary Figure S3. Pro1 is required for the response to cell wall stress. (A) Colony phenotypes of the indicated strains grown on YPD (3% yeast extract, 1% peptone, 2% dextrose and 1.5% agar) medium at the indicated temperatures (28 or 34°C) or at 28 °C in the presence of the indicated compounds. Plates were spot inoculated with the indicated number of microconidia, incubated for 3 days (28°C) or 4 days (34 °C) and imaged. (B) Quantitative analysis of the colony area (cm^2) was performed using the ImageJ software. The graph shows the values for the 104 colonies. Error bars represent standard deviations calculated from three replicate plates. Values with the same letter are not significantly different according to one-way ANOVA followed by Tukey's multiple comparison test ($P < 0.05$). CFW, Calcofluor white; CR, Congo red.

Supplementary Tables

Supplementary Table S1. Fungal Pro1 orthologues used for amino acid alignment.

Organism	Protein	GenBank Accession Number
Sordaria macrospora	PRO1	XP_003351793.1
Neurospora crassa	ADV-1	XP_958476
Cryphonectria parasitica	PRO1	ACI96308.1
Aspergillus nidulans	RosA	CAD58393.1
Aspergillus nidulans	NosA	CAJ76908.1
Aspergillus flavus	NosA	RMZ40455.1
Epichloë festucae	PROA	BAN17453.1
Fusarium oxysporum f. sp. *lycopersici*	Pro1	XP_018234693.1

Supplementary Table S2. Fungal strains used in this study.

Strain	Background	Genotype	Reference
4287	4287	Wild type	[29]
*mpk1*Δ	4287	Knockout mutant for the *mpk1* locus	[10]
*fmk1*Δ	4287	Knockout mutant for the *fmk1* locus	[29]
*pro1*Δ#9	4287	Knockout mutant for the *pro1* locus	This study
*pro1*Δ#23	4287	Knockout mutant for the *pro1* locus	This study
*pro1*Δ+pro1	pro1Δ#23	Complemented knockout mutant	This study
*pro1*Δ+*PgpdA-pro1-GFP*	pro1Δ#23	Constituve expression of *pro1* locus	This study
*fso1*Δ	4287	Knockout mutant for the *fso1* locus	[8]
*laeA*Δ	4287	Knockout mutant for the *laeA* locus	[39]
*veA*Δ	4287	Knockout mutant for the *veA* locus	[39]
*ste12*Δ	4287	Knockout mutant for the *ste12* locus	[7]

Supplementary Table S3. Oligonucleotides used in this study. Lowercase nucleotides were added to generate overlapping ends for fusion PCR.

Primer	Annealing Temp.	Nucleotide Sequence 5′ – 3′
Pro1 PF	62°C	AGGTGTAGAGGAGCTTCGATT
Pro1 PR	62°C	tttacccagaatgcacaggtacacttgtttaAGTGCAGCCATAGTCAGTTGT
Pro1 PFN	62°C	GTTGGATGGGGGATTTTAGTG
Pro1 TF	62°C	tggtcgttgtaggggctgtattaggtCTCGGAGAGGCACAACTGGGATAG
Pro1 TR	62°C	GGGCATAGAGTGAAAGTGTTG
Pro1 TRN	62°C	CTGGTTCTTTGTCCCTGCTG
Pro1 comp F	62°C	ACAACTGACTATGGCTGCACT
Pro1 comp R	62°C	ATGGTGTGATGATGCTGACCT
Pro1 comp R nest	62°C	CCCAGTTGTGCCTCTCGGAG
Clover Pro1	62°C	acaaggacgatgacgataagggtggttctATGTCTACGCTGCCCCCCGA
Pro1 Rev	62°C	AAAAGTCCCACCCCTTCGATT
GpdA15B	62°C	GGATCCCGAGACCTAATACAGCCCCT
GpdA15 nest	62°C	ACTAAATCGACTTCAGCAACA
TrpC8B	62°C	GGATCCAAACAAGTGTACCTGTGCATTC
Sv40 rev nest	62°C	CTAGATACCACATTTGTAGAGG
Gpda5	62°C	TCCAAGAACCTTTATTTCCCCT
Hyg-G	62°C	ACGTTGCAAGACCTGCCTGAA
Hyg-Y	62°C	GGATACCTCCGCTCGAAGTA
Act-q7	64°C	ATGTCACCACCTTCAACTCCA

Supplementary Table S4. Plasmids used in this study.

Plasmid	Origin/features	References
pAN7-1	Derived from pUC18; *A.nidulans gpdA* promoter; hygromicin B phosphotransferase gene from *Streptomyces spp.* (*hph*); *A. nidulans trpC* terminator	[36]
pUC57- 3xFomClover-3XFLAG	Derived from pUC57; *A. nidulans gpdA* promoter; 3 copies in tandem of the *mClover3* gene codon-adapted to *F. oxysporum* (3x*FomClover*); 3 copies of FLAG.	[46]
pUC57- 1xFomClover-3XFLAG	Derived from *pUC57- 3xFomClover- 3xFLAG.* Two of the three copies of *FomClover3* were removed.	This study.
NeoR	Derived from pAc5-STABLE2-Neo and pAN7-1 plasmids. *A. nidulans gpdA* promoter; neomycin resistance gene (*neo*); *A. nidulans trpC* terminator**.**	[37]

Supplementary Table S5. Statistical significance of tomato *Solanum lycopersicum* survival curves after inoculation with indicated *Fusarium oxysporum* strains, compared with the wild type. Data were analysed with the program GraphPad Prism version 8.0.1 and P values were calculated using the Log-rank (Mantel-Cox) test.

**Strain**	**P value**
*fmk1*Δ	<0,0001
*pro1*Δ#9	0,5131
*pro1*Δ#23	0,6655
*pro1*Δ#23+*pro1*	0,9106
*pro1*Δ#23+*PgpdA-pro1-GFP*	0,5916
H2O control	<0,0001
